# UV-A and UV-B combined with photosynthetically active radiation change plant growth, antioxidant capacity and essential oil composition of *Pelargonium graveolens*

**DOI:** 10.1186/s12870-023-04556-6

**Published:** 2023-11-10

**Authors:** Maryam Jadidi, Hasan Mumivand, Abdollah Ehtesham Nia, Alireza Shayganfar, Filippo Maggi

**Affiliations:** 1 Department of Horticultural Sciences, Faculty of Agriculture, Lorestan University, P.O. Box 465, Khorramabad, Iran; 2 Department of Horticultural Sciences and Landscape Engineering, Faculty of Agriculture, Malayer University, Malayer, 6586365719 Iran; 3 Chemistry Interdisciplinary Project (ChIP), School of Pharmacy, University of Camerino, Camerino, Italy

**Keywords:** PAR intensity, Ultraviolet A, Ultraviolet B, Essential oil, Citronellol

## Abstract

**Background:**

The different wavelengths of solar radiation incident on earth [herein: Photosynthetically Active Radiation (PAR) , Ultra Violet-A (UV-A) and Ultra Violet-B (UV-B)] and their spectral balance not only have an impact on plants’ growth, morphology and physiology, but also are important for the quality and quantity of plant secondary metabolites.

**Material and methods:**

In an outdoor study we addressed the effects of PAR intensity and UV-A and UV-B on the growth, yield, phenolic and flavonoid content, antioxidant activity and essential oil composition of *Pelargonium graveolens* L’Hér. The experiment was performed with split plots in a randomized complete block design with three replications. During the growth, two PAR intensities (ambient PAR and reduced PAR) and four UV treatments (ambient UV, enhanced UV-A, enhanced UV-B and enhanced UVA + B) were applied.

**Results:**

High PAR intensity decreased the length and width of leaf, the height of plant and fresh weight of aerial parts, and increased the dry weight of aerial parts. Enhanced UV-B irradiation was associated with reduced plant height, leaf expansion and fresh and dry weight of aerial parts. Interestingly, the negative effect of UV-B radiation on morphology and growth of plant was largely alleviated by high PAR intensity. The amount of total phenols and flavonoids, antioxidant activity and essential oil production of *P. graveolens* strongly increased with the increase of UV-B irradiation and PAR. On the other hand, UV-A radiation did not significantly influence total phenol and flavonoid content, antioxidant activity and essential oil composition. Moreover, the combination of high PAR intensity and UV-B led to further increases in total flavonoid content and antioxidant capacity. Both high PAR intensity and enhanced UV-B increased the percentage of geraniol in essential oil, leading to a slight reduction of citronellol/geraniol ratio which is a marker of quality for rose geranium essential oil.

**Conclusions:**

Overall, we conclude that UV-B irradiation was associated to reduction of plant growth and yield, while, the adverse effect of UV-B irradiation on the plant was mitigated by high PAR intensity. On the other hand, both high PAR and enhanced UV-B boosted the production of phenols, flavonoids and essential oil. Considering that the lower citronellol/geraniol ratio is the most important indicator for the economic value of rose geranium essential oil, reducing citronellol/geraniol ratio under enhanced UV-B radiation and/or high PAR is likely to be favorable.

## Background

Since the human activities have changed the Earth's atmosphere and consequently depleted the ozone layer, the investigation on the biological effects of ultraviolet (UV) irradiation has become an interesting issue in recent decades. The UV irradiation consists of a minor portion (8–9%) of the full solar irradiation [1, 2]. This radiation, as an important environmental factor, induces diverse adaptive changes during life cycle and could affect the adaptive mechanisms of plants to known stressor factors [3]. UV radiation traditionally is divided into three parts based on the wavelength: UV-A (320–400 nm), UV-B (280–320 nm), and UV-C (200–280 nm) radiations. In plant systems each band can induce significantly different biological effects [4]. UV-C quickly causes serious damage to cellular components [5]. UV-C radiation is effectively absorbed by ozone and ambient gasses, and is not present at ground level at sunlight. UV-B irradiation has pleiotropic influences on the plant growth, development, biochemical response, physiology and morphology [6, 7]. It acts as an eco-physiological factor that is capable of changing plant metabolism by switching between primary and secondary metabolites [8]. The UV-B radiation produces reactive oxygen species (ROS) which damage cell membranes, proteins, DNA, and delay photosynthesis with reduction of the plant growth [9–11]. UV-A radiation comprises a much higher proportion of the solar UV spectrum (up to 95% near the equator) [12, 13]. This part of solar UV passes almost unaltered through the atmosphere and is capable of penetrating the inner tissues. However, it is considered less harmful than UV-B [14, 15]. Unlike UV-B, seasonal variation and different times of the day have little effect on daily UV-A flux [12, 13, 16]. UV-A can cause oxidative damage and growth inhibition in higher plants [17, 18]. On the other hand, UV-A radiation is reported to mitigate the damaging influences of UV-B [19, 20]. The increased levels of pigments and UV-absorbing compounds such as flavonoids, carotenoids, and other antioxidants have been reported to enhance growth by supplementation of UV-A and photosynthetically active radiation (PAR: 400–700 nm) [20–22].

In higher plants, light not only is an energy source but also acts as a key signal regulating growth, development and metabolism [23]. On the other hand, the spectral balance between photosynthetically active radiation (PAR) intensity, UV-A, and UV-B has been demonstrated to influence plant response to UV irradiation [17]. Plants have evolved a number of defense systems to cope with UV-B stress factors in nature. As an avoidance strategy, exposure of plants to UV-B radiation can result in increasing biosynthesis of antioxidant compounds such as phenolics and carotenoids, as well as other natural products [24, 25]. PAR intensity has also remarkable effects on plant growth, development and secondary metabolite biosynthesis. Thus, plant response to the adverse effects of high PAR intensity and UV-B irradiation may overlap, imposing cross tolerance to both parts of sunlight [26–28].

Currently, attenuation and enhancement in field experiments are two of the most suitable approaches used to investigate plant responses to UV and PAR. Field attenuation studies are the most applicable from a spectral perspective, as plants are exposed to balanced ratios of UV/PAR which is greatly modifiable in greenhouse and growth chamber studies. Hence, supplementary UV-A and UV-B studies allow to know the influence of UV irradiation and generate realistic data on the biological effects of UV-B and/or UV-A irradiation on development and growth of individual plants, plant communities or whole ecosystems [13]. Moreover, these experiments can give realistic sensitivity assessments and acclimation of plants to ambient UV-B and UV-A levels.

Rose geranium (*Pelargonium graveolens* L’Hér.), a perennial aromatic plant belonging to the family of Geraniaceae, is mostly cultivated for its essential oil which is economically valuable [29]. Rose geranium oil, obtained from the green foliage and flowers of the plant, finds extensive application in cosmetics, food, perfumery, and pharmaceutical industries [30, 31]. The essential oil is traditionally used as antioxidant, antibacterial, antifungal, antidiabetic, antiallergic, antidiarrhoeic, antihepatotoxic, anti-inflammatory, anti-spasmolytic, diuretic, and tonic [32, 33]. In aromatherapy, it is used as remedy for menopausal problems, nervous tension and anxiety, and as a skincare agent [30]. The chemical composition of rose geranium oil has been the subject of several studies. Citronellol, geraniol, linalool, citronellyl formate and *p*-menthone were found to be the major components of the essential oil [29, 34–36].

Plant growth and morphology as well as essential oil production and constancy are strongly affected by the combined effects of the genetic factors and the environmental conditions [37]. Indeed, even though the essential oil content and composition is primarily under genetic control, its production is also particularly dependent on environmental factors including light intensity, UV irradiation, irrigation, nutrition, day length, temperature and drying method [38–41]. Therefore, the influence of PAR intensity and UV irradiation on essential oil production in rose geranium could have useful implications on management of product quality and standardization in open field cultivation. Hence, the present study aimed to investigate growth and yield, essential oil production, total phenol and flavonoid content as well as antioxidant activity of *P. graveolens* exposed to different PAR intensity and UV-A and UV-B radiation. Indeed, an attempt was made to illuminate whether high PAR intensity could potentially minimize the detrimental effects of UV irradiation on the growth and yield of rose geranium.

## Materials and methods

### Plant materials and experimental site

The experiment was carried out from early June to late September in the years 2020 and 2021. Stock plants of *P. graveolens* were obtained from Medicinal Plants Garden of Lorestan University, Lorestan, Iran. Healthy cuttings were collected from the mother plant and placed in plastic pots with a substrate of sand and kept in the greenhouse. Plants were raised under conditions of natural PAR irradiances (up to 600 μmol photons/m^2^/s) and exclusion of UV-A and UV-B for 30 days. After root formation, the cuttings were transplanted into 8 L pots (one plant in each pot) containing a substrate of soil, sand, and cow manure (1:1:1) as substrate. Then, rose geranium plants were transferred to the outside of greenhouse at the beginning of June. Acclimatization of plants to the conditions of open air was done two weeks prior to the PAR and UV treatments. The outdoor setting was conducted in the research garden of Lorestan University of Khorramabad (longitude 48°26’ E, latitude 33°44’ N, altitude 1170 m). During the experiment, plants were irrigated twice a week and fertilized with humic acid (2.5 mL/L) twice.

### UV and PAR treatments

The experiment was done as split plots in a randomized complete block design with three replications. Two PAR intensities (ambient PAR and reduced PAR) (as main plots) were applied, as well as four UV treatments (solar ambient UV, enhanced UV-A, enhanced UV-B, and enhanced UVA + B) (as subplots). Application of different levels of PAR and UV was started two weeks after transferring the pots to the outdoor. The rose geranium plants (5 pots per each treatment) received the individual treatments for three months. At the beginning of treatments, photoperiod was 14:30 h/9:30 h with day/night. In order to evaluate whether UV irradiation influences on rose geranium depending on PAR intensity, plants were exposed to two different PAR intensities. Average of ambient PAR intensity (High PAR) was at 1500 mmol/m^2^/s during midday on sunny days. In reduced PAR treatment (low PAR), plants received the PAR intensity of 800 mmol/m^2^/s by shading (corresponded to 53% of natural PAR intensity).

**Fig. 1:**
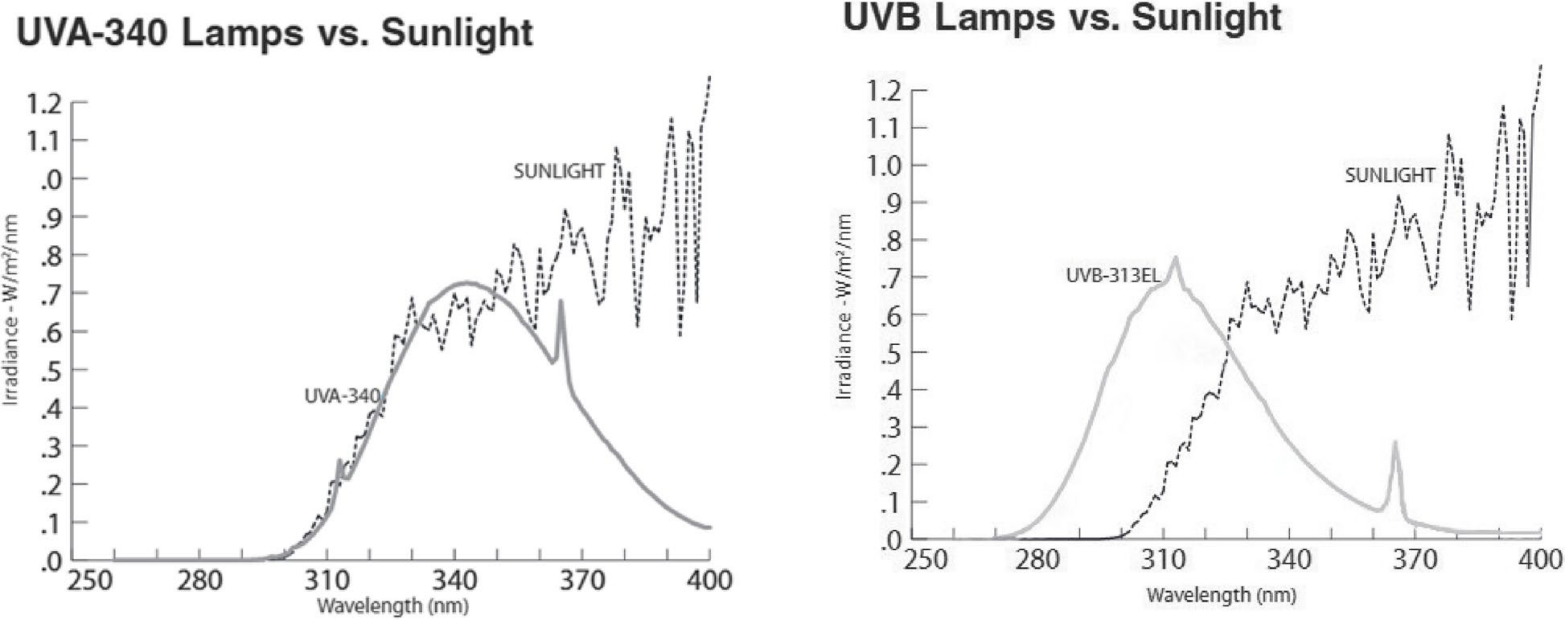
The spectral irradiance emitted by the employed UVA and UVB lamps, as compared to that of sunlight assessed on a clear day at solar noon

In enhanced UV-B and UV-A plots, the supplementary UV-B and UV-A treatments were applied in late June until the full flowering phase in late September. Plants were artificially subjected to UV-A and UV-B radiations via UV-A lamps (Q panel UV-A 340 40 W, 120 cm lamps, Q Panel Inc. Cleveland, OH, USA) and UV-B lamps (Q panel UV-B 313 40 W, 120 cm lamps, Q Panel Inc. Cleveland, OH, USA), respectively (Fig. 1) [42]. In order to reduce shading of fluorescent lamps and metal frames, the experimental plots were placed in orientation of east–west. The lamps were fixed on frameworks at the northern margin of plots and UV radiation was reflected on the plants. The lamps were placed in steel frames with a distance of 25 cm between lamps at a distance of about 50 cm with plants. This distance was maintained steady until the experiment was virtually over. In the UV-A and UV-B plots, plants received ambient plus supplemental levels of UV-A and UV-B, respectively. The mean of UV-B_BE_ (biologically effective UV-B radiation) and UV_BE_ (biologically effective UV radiation) daily doses reaching the plants under ambient UV treatments were 8.98 and 14.5 kJ m^−2^ day^−1^, respectively. While, plants exposed to UV-B lamps received ambient UV-B_BE_ + 3.8 kJ m^−2^ d^−1^ UV-B. The mean of UV_BE_ daily doses received by the plants under UV-A irradiation was ambient UV_BE_ + 11.7 kJ m_−2_ day^−1^. Enhanced UV treatments were given to the plants at midday (11:00–14:00 h) by electric timer. The UV lamps were wrapped in cellulose diacetate foil to block residual UV-C irradiation (< 280 nm). In ambient UV plots, plants were exposed to ambient rates of solar UV radiation.

### Evaluation of biomass and yield attributes

At the full flowering stage, biomass and yield attributes including plant height, leaf length, leaf width, fresh and dry weight of plant aerial parts and dry weight of leaves were measured. Plants were harvested and instantly weighed (recorded fresh weight), followed by 72 h of oven drying at 50 °C and reweighing (recorded dry weight). The oil-bearing tissue of the plant (leaves) were detached from the woody parts, weighed and noted as the leaf dry weight of *P. graveolens*.

### Total phenol content

The total phenol content was assayed by Folin-Ciocalteu reagent method [43]. First, 1 g of dried leaves was ground and extracted in 10 mL of methanol (80%) and incubated at 20–25 °C for 48 h in dark. After filtering the extract, 0.25 mL of extract, 0.25 mL Folin Ciocalteu reagent, and 2 mL Na_2_CO_3_ (1 M) were mixed well and left to stand at room temperature for 15 min. Finally, the absorbance of all samples was recorded using a spectrophotometer at 765 nm. All samples were analysed three times with the total phenol concentration measured based on the gallic acid standard curve prepared using 0, 50, 100, 150, 250, and 500 mg/L solutions. The total phenol content was expressed in gallic acid equivalent (mg GAE/g dry weight).

### Total flavonoid content

The total flavonoid content was determined using the colorimetric method of aluminum chloride [44]. Fifty µL of the standard or extract, 400 µL of 2% AlCl_3_, and 1200 µL of 5% CH_3_COOK were mixed and kept at 22 °C for 40 min at 37 °C. The absorbance of samples was read at 415 nm using a spectrophotometer. The standard curve was prepared using solution of rutin with concentrations of 0, 50, 100, 150, 250 and 500 mg/L and the total flavonoid content was expressed in rutin equivalent (mg rutin/100 g dry weight). The number of repetitions was three.

### Antioxidant activity

The antioxidant capacity of leaf extracts was determined by DPPH (2,2-dipheny-l-picrylhydrazyl) assay as in Choi et al. [45]. Various solutions of each extract (dosage range of 0.01–1.0 mg/mL) were prepared in methanol. Afterwards, 1.0 mL of DPPH solution 3.0 × 10^–4^ M and 2.5 mL of the extract were vigorously mixed and incubated in the dark at 20 °C for 30 min. The absorbance of samples was read at 517 nm using a spectrophotometer. Then, the absorbance of a blank containing 2.5 mL of plant extract and 1.0 mL methanol was also read. Three repetitions were measured for each sample. Finally, the radical scavenging activity of each sample was expressed based on IC_50_ (The half maximal inhibitory concentration).

### Essential oil isolation

Twenty-five g of dried leaves were separately subjected to hydro-distillation using a Clevenger-type apparatus over 3 h. The essential oil was gathered in a glass vial, then stored at 4 °C until GC-FID and GC–MS analyses. The content of essential oils obtained from each treatment was calculated from three sequential repetitions and expressed in mg per 100 g dry weight (w/w) [40].

### GC–MS and GC–FID analyses

GC–MS and GC-FID analyses of essential oils were performed according to the work of Mumivand et al. [46]. For GC–MS analysis, a Shimadzu gas chromatograph coupled with a mass spectrometer was employed. Essential oil constituents were separated through a BP-5 fused-silica capillary column (Shimadzu) of 30 m × 0.22 mm i.d. coated with 0.25 μm film. Carrier gas: ultra-pure helium; injector and interface temperatures: 280 and 260℃, respectively; ionization voltage:70 eV; split ratio: 1/20; ion source and transfer-line temperatures: 250 °C; mass spectrum: 35–450 amu. The oven temperature was adjusted to rise from 50 to 280 °C at a rate of 5 °C/min and finally held isothermally for 15 min.

The GC-FID analysis of essential oils was done through a Thermoquest Finnigan apparatus equipped with FID detector and a fused-silica capillary column of BP-5 with 30 m l. × 0.25 mm i.d. and film thickness of 0.25 mm. The temperature of oven was similar to GC–MS; detector and injector temperatures: 300 °C and 250 °C, respectively; carrier gas: helium; flow rate: 1 ml/min; split ratio: 1/20. Retention indices of each oil constituent were calculated using a homologous series of *n*-alkanes (C_5_-C_25_) injected into BP-5 column using the same analytical conditions. Constituents of essential oils were identified by comparing (1) the retention time with those from available authentic standards, (2) the mass spectra with those of the internal library, and (3) the retention indices (KI) with those presented in the literature. Relative area percentage obtained by GC–FID for each peak was used for quantification, without considering response factors [46].

### Data analysis

The mean of data obtained from the 2-years study was subjected to variance analysis according to the experimental design by SAS 9.1.3 (SAS Institute Inc.), and mean comparison was done using Duncan's multiple range test at *P* ≤ 0.05.

## Results

### Biomass and yield attributes

Results of the present study revealed that the length and width of leaf were significantly increased by lowering PAR intensity. The highest leaf length (5.37 cm) and leaf width (7.86 cm) were observed in plants exposed to low PAR intensity. On the contrary, increasing PAR intensity significantly improved the leaf dry weight. Leaf dry weight was increased by 21.95% when plants were exposed to high PAR compared with low PAR (Table 1).

**Table 1:** The effect of different levels of PAR intensity on yield attributes, total phenol and essential oil content and yield of *P. graveolens*

PAR	Leaf length (cm)	Leaf width (cm)	Leaf dry weight (g)	Total phenol (mg Gallic acid/g dry weight)	Essential oil content (%)	Essential oil yield (g/plant)
High PAR	3.88^b^	5.69^b^	69.78^a^	68.54^a^	0.431^a^	0.298^a^
Low PAR	5.37^a^	7.86^a^	57.22^b^	55.91^b^	0.336^b^	0.187^b^

Means with similar letters in each column, based on Duncan test at a 0.05% probability level, do not differ significantly

^a,b^show a significant difference between experimental treatments

The length and width of leaf were significantly decreased by UV-B treatment in comparison with ambient UV. However, we did not find any effects of the UV-A irradiation on leaf dimensions. The highest leaf length and width was observed with ambient UV treatment (5.48 and 7.94 cm, respectively). Furthermore, leaf dry weight was significantly reduced by UV-B treatment. The highest leaf dry weight (69.4 g) was obtained with ambient UV treatment (Table 2).

**Table 2:** The effect of UV irradiation on yield attributes, total phenol and essential oil content and yield of *P. graveolens*

UV irradiation	Leaf length (cm)	Leaf width (cm)	Leaf dry weight (g)	Total phenol (mg Gallic acid/g dry weight)	Essential oil content (%)	Essential oil yield (g/plant)
Ambient UV	5.48^a^	7.94^a^	69.41^a^	55.52^c^	0.283^b^	0.192^b^
Enhanced UVA	5.4^a^	7.57^a^	67.22^a^	55.23^c^	0.321^b^	0.225^b^
Enhanced UVB	3.82^b^	5.85^b^	57.55^b^	66.88^b^	0.467^a^	0.277^a^
Enhanced UVA + B	3.81^b^	5.73^b^	59.83^b^	71.28^a^	0.464^a^	0.276^a^

Means with similar letters in each column, based on Duncan test at a 0.05% probability level, do not differ significantly

^a,b^show a significant difference between experimental treatments

The interaction effect of UV radiation * PAR intensity was significant on height of plant and fresh and dry weight of aerial parts. The highest plant height (51.08 cm) and fresh weight of aerial parts (579 g) were observed in plants exposed to low PAR intensity and UV-A irradiation. On the contrary, the highest dry weight was obtained when plants were subjected to enhanced UV-A irradiation and high PAR intensity (Table 3).

**Table 3:** The interaction effect of PAR intensity and UV irradiation on yield attributes, total flavonoid and antioxidant capacity of *P. graveolens*

PAR intensity	UV irradiation	Plant height (cm)	Plant fresh weight (g)	Plant dry weight (g)	Total flavonoids (mg Routin/g dry weight)	Antioxidants capacity (μg/mL)
High PAR	Ambient UV	38.67^bc^	512^c^	95.94^ab^	19.69^c^	0.082^b^
	Enhanced UVA	40^b^	544^b^	101.54^a^	20.2^c^	0.066^bc^
	Enhanced UVB	30.67^d^	437^d^	90.91^b^	23.77^b^	0.052^c^
	Enhanced UVA + B	32.5^d^	441^d^	95.85^ab^	28.17^a^	0.051^c^
Low PAR	Ambient UV	46.67^a^	572^a^	91.85^b^	13.97^d^	0.16^a^
	Enhanced UVA	51.08^a^	579^a^	95.58^ab^	15.08^d^	0.143^a^
	Enhanced UVB	32.07^d^	314^f^	58.15^c^	24.78^b^	0.083^b^
	Enhanced UVA + B	33.67^ cd^	384^e^	61.98^c^	26.1^ab^	0.068^bc^

Means with similar letters in each column, based on Duncan test at a 0.05% probability level, do not differ significantly

^a,b,c,d,e,f^show a significant difference between experimental treatments

^ab, bc, cd^indicate a statistically significant difference between those groups

### Total phenol content, total flavonoid content and antioxidant activity

The concentration of total phenolics was significantly enhanced following the increasing PAR intensity. Total phenol content was increased by 22.59% in the high PAR treatment as compared with the low PAR (Table 1). Furthermore, UV-B irradiation strongly increased the total phenol content in comparison with ambient UV treatment. On the other hand, we did not observe any effects of the UV-A irradiation on the total phenol content. Exposure of plants to the UV-A + B irradiation resulted in the highest total phenol content (71.28 mg GAE/g dw) (Table 2). Interaction of PAR intensity * UV treatments was significant for the total flavonoid content. The highest amount of total flavonoid content (28.17 mg rutin per g dry weight) was obtained with high PAR intensity and UV-A + B treatment. The results indicated that the antioxidant capacity of plants was strongly affected by the interaction of PAR intensity * UV treatments. The lowest IC_50_ value (0.05 μg/mL) was obtained when plants were exposed to high PAR intensity and UV-A + B irradiation (Table 3).

### Essential oil content and yield

The results revealed that under high PAR intensity, the essential oil content and yield were increased by 28.27 and 59.35%, respectively, when compared with the low PAR intensity. The highest essential oil content (0.431%) and yield (0.298 g/plant) were obtained when plants were exposed to high PAR intensity (Table 1). The essential oil content and yield under UV-B irradiation showed a significant increment, so that the highest values (0.467% and 0.277 g/plant, respectively) were obtained under enhanced UV-B treatment (Table 2).

### Essential oil composition

**Fig. 2:**
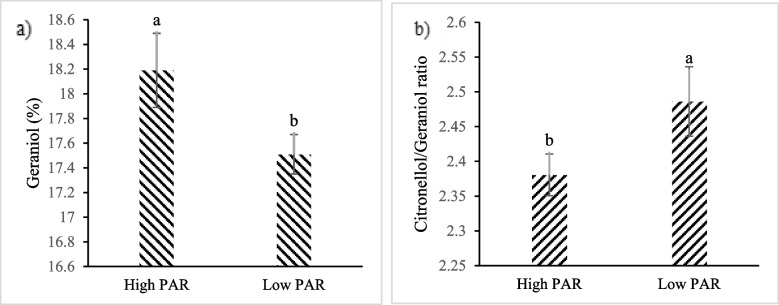
The effect of different levels of PAR intensity on **a** geraniol and **b** citronellol/geraniol ratio in the essential oil of *P. graveolens*

**Fig. 3:**
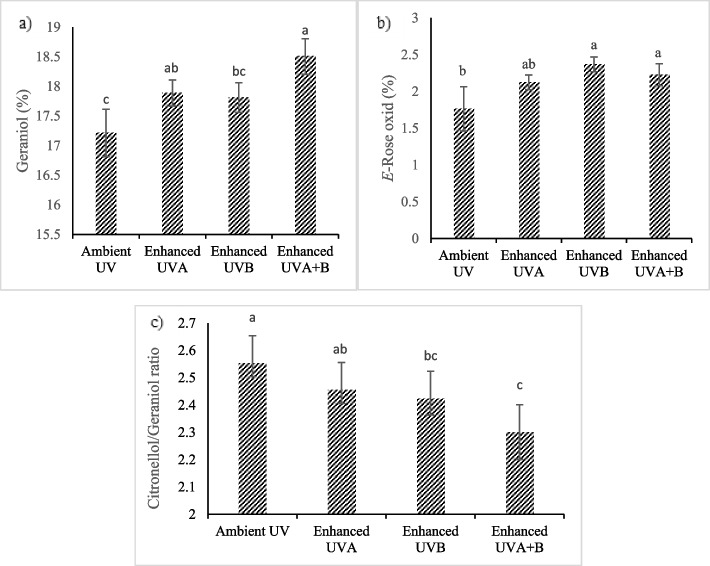
The effect of UV irradiation on **a** geraniol, **b** *E*-rose oxide and **c** citronellol/geraniol ratio in the essential oil of *P. graveolens*

In the present study, 34 constituents were identified in the essential oil of *P. graveolens* using GC–MS and GC-FID analysis (Table 4). Citronellol (up to 46.28%) and geraniol (up to 18.5%) were detected as the most abundant components. Other notable constituents were neral (up to 4.3%), geranyl formate (up to 3.6%), *E*-rose oxide (up to 2.4%), citronellyl formate (up to 2%), *β*-elemene (up to 1.6%) and caryophyllene oxide (up to 1.5%). The mean comparison revealed that the percentage of geraniol in essential oil was enhanced by increasing the PAR intensity. The highest amount of geraniol (18.19%) was obtained in plants exposed to high PAR intensity. The citronellol/geraniol (C/G) ratio was significantly reduced from 2.49 in low PAR intensity to 2.38 in high PAR intensity (Fig. 2). UV-B irradiation significantly induced *E*-rose oxide production, so that the highest amount of *E*-rose oxide (2.37%) was noted under enriched UV-B treatment. The highest total amount of geraniol was observed in plants treated with both UV-A and UV-B. On the contrary, citronellol / geraniol ratio was significantly reduced with UV-B irradiation. The highest citronellol / geraniol ratio was shown with ambient UVA treatment (Fig. 3).

**Table 4:** Essential oil constituents of *P. graveolens*

NO	Oil Constituents	RI^a^	LIT RI^b^	ID^c^
1	*p*-Cymene	1018.44	1020	RI, MS
2	Limonene	1020.15	1024	Std
3	*β*-*E*-Ocimene	1042.37	1044	RI, MS
4	Linalool	1090.95	1095	Std
5	*Z*-Rose Oxide	1102.55	1106	RI, MS
6	*E*-Rose Oxide	1120.76	1122	RI, MS
7	*β*-Citronellal	1142.86	1148	RI, MS
8	*Z*-*p*-Menthan-2-one	1190.3	1194	RI, MS
9	Citronellol	1122	1223	Std
10	Neral	130.32	1235	RI, MS
11	Geraniol	1249.64	1249	Std
12	Geranial	1261.65	1264	RI, MS
13	Neryl formate	1275.27	1280	RI, MS
14	Citronellyl formate	1270.88	1271	RI, MS
15	Geranyl formate	1288.64	1298	RI, MS
16	Methyl geranate	1320.45	1322	RI, MS
17	2-Phenyl ethyl propanoate	1350.65	1351	RI, MS
18	*α*-Cubebene	1352.64	1345	RI, MS
19	Citronellyl acetate	1353.96	1350	RI, MS
20	*α*-Copaene	1370.34	1374	RI, MS
21	*β*-Elemene	1385.65	1389	Std
22	*β*-Bourbonene	1386.7	1387	RI, MS
23	*E*-Caryophyllene	1409.64	1417	Std
24	Aromadendrene	1436.5	1439	RI, MS
25	Citronellyl propanoate	1447.65	1444	RI, MS
26	Germacrene-D	1482.5	1484	Std
27	Leden	1493.8	1496	RI, MS
28	Citronellyl butyrate	1513.5	1517	RI, MS
29	*E*-Calamenene	1522.4	1528	RI, MS
30	Geranyl butyrate	1558.42	1559	RI, MS
31	Spathulenol	1574.62	1577	Std
32	Caryophyllene oxide	1581.43	1582	Std
33	Phenyl ethyl tyglate	1583.54	1584	RI, MS
34	*α*-Bisabolol	1682.3	1685	RI, MS

^a^RI, linear retention indices on HP-5 column, experimentally determined using homologue series of n-alkanes

^b^Relative retention indices taken from Adams

^c^Identification methods: MS, by comparison of the mass spectrum with those of the computer mass libraries Wiley, Adams and NIST 05; RI: by comparison of retention index with those reported in literature; Std: by comparison of the retention time and mass spectrum of available authentic standard

**Fig. 4:**
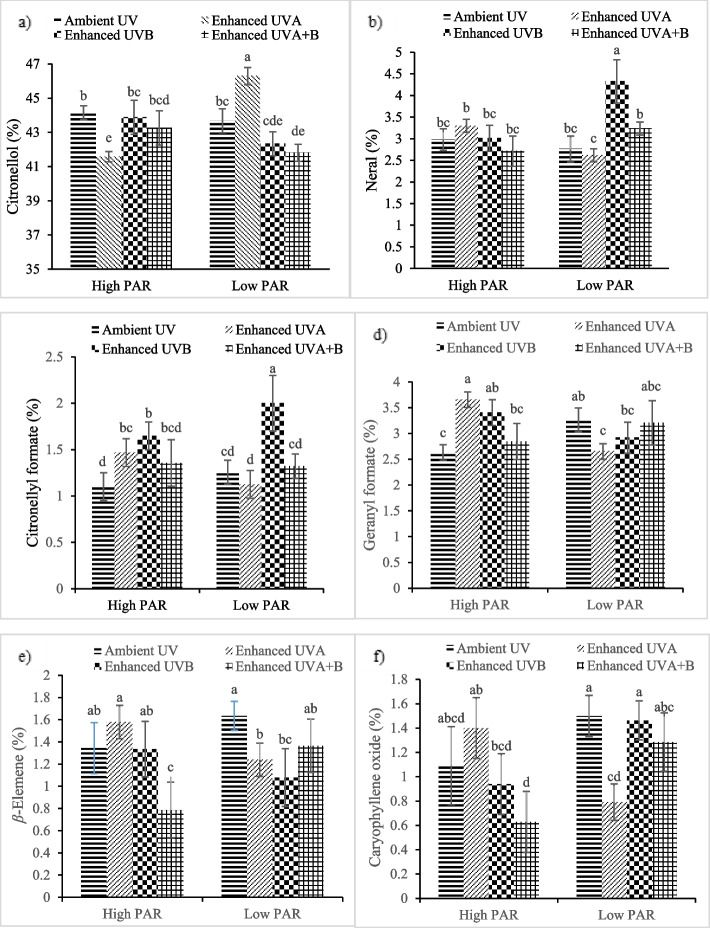
The interaction effect of PAR intensity and UV irradiation on **a** citronellol, **b** neral, **c** citronellyl formate, **d** geranyl formate, **e** *β*-Elemene and **f** caryophyllene oxide in the essential oil of *P. graveolens*

By comparing the mean interaction of UV irradiation * PAR intensity, the highest percentage of citronellol (46.28%) as the main essential oil ingredient was obtained under both low PAR intensity and enhanced UVA treatment. The highest percentage of neral (4.32%) and citronellyl formate (2%) were found when plants were exposed to both low PAR intensity and enhanced UV-B. During subsequent exposure to high PAR intensity and UV-A, plants showed the highest percentage of geranyl formate (3.65%). Furthermore, the highest percentages of *β*-elemene (1.63%) and caryophyllene oxide (1.5%) were noted under low PAR intensity and ambient UV (Fig. 4).

### Correlation between traits

The essential oil content showed a negative correlation with plant height (-0.74**) and plant fresh weight (-0.61**). On the other hand, it was positively correlated with total phenol (0.82**) and flavonoid (0.79**) contents. There was a high positive correlation between essential oil yield and essential oil content (0.88**). The correlation coefficient between antioxidant activity with essential oil content and yield, total phenol, flavonoid and citronellol/geraniol ratio were calculated as 0.82**, 0.79**, 0.75** and 0.75**,0.67**, respectively. The percentage of geraniol in essential oil was negatively correlated with the plant height (-0.66**), plant fresh weight (-0.60**), plant dry weight (-0.55**) and leaf dry weight (-0.40*). Finally, citronellol/geraniol ratio correlation with essential oil content (0.87**), essential oil yield (0.69**), phenol (0.51*) and flavonoid (0.74**) was positive (Table 5).

**Table 5:** The correlation coefficients between yield attributes, antioxidant activity, phenol, flavonoid, essential oil content and yield and major compounds of essential oil of *P. graveolens*

	Height	PFW	PDW	LDW	Oil content	Oil yield	Phenol	Flavonoid	DPPH	Citronellol	Geraniol	C/G ratio
Height	1											
PFW^a^	0.78^**^	1										
PDW^b^	0.39	0.81^**^	1									
LDW^c^	0.21	0.68^**^	0.94^**^	1								
Oil content	-0.74^**^	-0.61^**^	-0.19	-0.12	1							
Oil yield	-0.59^**^	-0.25	0.26	0.36	0.88^**^	1						
Phenol	-0.72^**^	-0.45^*^	0.013	0.12	0.82^**^	0.83^**^	1					
Flavonoid	-0.82^**^	-0.77^**^	-0.41^*^	-0.28	0.79^**^	0.62^**^	0.79^**^	1				
DPPH	-0.77^**^	-0.55^**^	-0.12	0.07	0.75^**^	0.75^**^	0.79^**^	0.82^**^	1			
Citronellol	-0.07	0.12	0.36	0.28	0.12	0.21	0.14	0.15	0.05	1		
Geraniol	-0.40^*^	-0.55^**^	-0.60^**^	-0.66^**^	0.07	-0.23	0.02	0.22	-0.05	-0.02	1	
C/G ratio	-0.79^**^	-0.77^**^	-0.49^*^	-0.39	0.74^**^	0.51^*^	0.69^**^	0.87^**^	0.67^**^	-0.01	0.31	1

*Significant at 5%

**Significant at 1% level

^a^Plant fresh weight

^b^Plant dry weight

^c^Leaf dry weight

## Discussion

In the present research, we studied the response of *P. graveolens* to UV and PAR in terms of growth, yield, phenol and flavonoid contents, antioxidant capacity and essential oil composition. Our results showed that high PAR intensity reduced the length and width of leaf and the height of plant, resulting in formation of dense and dwarf plants. Similar findings have been previously reported for *Dianthus caryophyllus* L. [47], *Hordeum vulgare* L. [48], *Pelargonium zonale* (L.) L'Hér. ex Aiton [10] *Rosmarinus officinalis* L. [23], which showed lower leaf area and height in the presence of high PAR intensity. However, similar to our results, plant dry weight was positively affected by PAR induction. As reported in previous studies, the high PAR intensity generally increased plant dry matter, and this effect is probably linked to the induced photosynthetic activity resulting in increased carbon fixation [23]. On the other hand, the increase of leaf area in plants exposed to low PAR intensity can be interpreted as a kind of adaptation strategy to maximize the capture of radiant energy [49].

Our results showed different growth responses between plants exposed to enriched UV-A and enriched UV-B, as enriched UV-A radiation was more compatible and favorable for *P. graveolens*. Enriched UV-B irradiation was associated with reduced plant height, leaf expansion and fresh and dry weight of aerial parts. These findings are in accordance with previous reported studies on UV-A and UV-B effects [11, 18]. Reduction in growth and yield of plants exposed to high UV-B irradiation is mostly due to the negative effects of UV-B on photosynthesis (especially photochemical efficiency of PSII) and chloroplast ultrastructure, DNA and photo-oxidative destruction [24, 48, 50]. Moreover, reduction of growth and yield of plants with UV-B irradiation was stronger under low PAR intensity than high PAR intensity, confirming that UV-B irradiation and high PAR synergistically reinforce defense systems in plant. Alike, it has been previously demonstrated that blue light may ameliorate UV-B adverse effects [10]. Similar findings have been previously published for two barley varieties, where negative influences of UV-B radiation on morphology and growth were largely alleviated by high PAR intensity [48]. As noted in that study, high PAR intensity reduced the negative influence of UV-B on the extent of light absorption, carboxylation activity and photochemical efficiency.

Increasing evidence suggests that exposure to excessive amounts of UV-B radiation quite often leads to overproduction of reactive oxygen species (ROS) in plant cells and to the impairing of the photosynthetic machinery [11]. Plant cells are typically safeguarded against the detrimental effects of ROS through the coordinated efforts of enzymatic and non-enzymatic antioxidant constituents. These antioxidant compounds work synergistically to effectively scavenge the increased ROS and alleviate stress [51]. Previous studies reported that UV-B promotes the activities of antioxidant compounds such as *α*-tocopherol, flavonoids, carotenoids and phenolics, which play important roles as ROS scavengers [9, 48, 52]. As noted in the present study, the amount of total phenols and flavonoids and antioxidant activity of *P. graveolens* strongly increased with the UV-B radiation. Of all groups of secondary metabolites, phenolic compounds, especially flavonoids, are considered the most important ones for UV-B defense. Therefore, it can be concluded that planst produce higher amount of these secondary metabolites to counteract the effects of UV-B irradiation. On the contrary, UV-A radiation did neither significantly influence total phenol and flavonoid contents nor antioxidant activity. Results demonstrated that high PAR intensity also increased the contents of total phenol and flavonoids in plant irrespective of UV treatment. Moreover, the combination of high PAR intensity and UV-B led to an increase in total flavonoid content and antioxidant capacity. Therefore, it was found that PAR and UV-B had an increasable influence on the accumulation of flavonoids as UV-absorbing compounds.

It is commonly acknowledged that epidermally located flavonoids, as photoprotective pigments, reduce the penetration of short wavelengths of solar radiation (280–450 nm) into leaves [53]. Thus, they efficiently protect the photosynthetic apparatus from high PAR intensity and UV-B irradiation and prevent photo-oxidative damage [52]. It has also been reported that flavonoids are endogenous regulators of transport and oxidation rates of indole acetic acid [54]. Therefore, further increase in flavonoid accumulation by UV-B and PAR irradiance could be attributed to their roles as defense signal compounds. Furthermore, our findings demonstrated the negative relationship between flavonoids concentration and growth and yield of the plant.

We have shown that both high PAR and UV-B particularly increased the essential oil content and yield of *P. graveolens.* In accordance with this observation, an increased essential oil content of the plants exposed to high PAR intensity has been reported in *Aeollanthus suaveolens* Mart. ex Spreng. [55], *R. officinalis* [23], *Eclipta alba* (L.) Hassk. [56], and has been commonly ascribed to the increased photosynthetic activity and secondary metabolism. The higher accumulation of plant secondary metabolites in response to high PAR intensity could also be explained by the ecological function of these compounds against high light intensity [23]. Differently from what observed in this research, shading of solar radiation has been associated to the increased essential oil content of some species, due to the protection conferred by shading against very intense radiation stress [57, 58].

The essential oil yield of oil-bearing plants is thoroughly dependent on the plant biomass and percentage of essential oil [59]. The extent of the increase of the essential oil content due to UV-B irradiation was higher than that of the decrease of the leaf dry weight. For this reason, the essential oil yield of the plant was increased with UV-B treatment. Our results are in accordance with observations of previous reports on other medicinal plants such as *Acorus calamus* L. [60], *Ocimum sanctum* L. [61], *Thymus* spp. [11] and *E. alba* [56]. Rai and Agrawal [56] reported that the content of *E. alba* essential oil was increased under continuous UV-B irradiation, and reduced under intermittent UVB irradiation. Kumari and Agrawal [61] have concluded that UV-B exposure is a substantial need for the development of the oil glands in *O. sanctum,* resulting in the boosting of essential oil secretion. One of the roles of essential oils is the participation in defensive mechanisms in response to environmental stresses. Thus, their production is associated with abiotic factors such as nutrient deficiency, salt stress, water scarcity, temperature, light intensity, and UV irradiation [39, 62]. Thus, increasing the essential oil content of rose-geranium to induce plant tolerance to UV-B irradiation and its oxidative stress seems reasonable. In this study, the increase of UV-A irradiation made no change in essential oil content and yield of plant. This result was inconsistent with finding reported by Mumivand et al. [18] who mentioned a positive effect of UV-A irradiation on the essential oil production of thyme. These differences in the responses of the plants could be due to factors including the inherent characteristics of the species, plant origin, climatic conditions, extraction methods, storage conditions, and dose and duration of UV-B irradiation [18, 63].

A change in essential oil composition under both UV-B irradiance and PAR intensity has been demonstrated in a majority of papers, although in some cases it remains constant. In the present study, the data showed that the UV irradiation and PAR intensity significantly affected the essential oil constituents of *P. graveolens.* High PAR intensity increased the percentage of geraniol in essential oil, leading to a slight reduction of citronellol/geraniol ratio. Furthermore, significant decrease in citronellol/geraniol ratio was observed under UV-B irradiation in comparison with ambient UV. The citronellol/geraniol ratio is a key index characterizing the quality of the essential oil in rose-geranium for the perfume industry [64]. Citronellol/geraniol ratio of 1:1–3:1 gives the economic value to rose-geranium essential oil [65]. However, it is believed that essential oils with a citronellol/geraniol ratio higher than 3:1 have poor quality for the perfume industry [66]. Considering that the lower citronellol/geraniol ratio is the most important indicator reduction citronellol/geraniol ratio under UV-B radiation and/or high PAR is likely to be favorable. 

Significant increase in the percentage of geraniol was observed under UV-B treatment. While, under low PAR intensity, neral and citronellyl formate percentages showed increase in plants exposed to enhanced UV-B. On the other hand, under high PAR intensity, caryophyllene oxide showed reduction under UV-B exposure. Citronellol and *β*-elemene found to be reduced in *P. graveolens* essential oil when plants were exposed to UV-B. On the contrary, under low PAR intensity, UV-A radiation was positive for high citronellol content. It is hypothesized that such modification in secondary metabolites biosynthesis is necessary for plant adaptation to biotic and abiotic stresses such as PAR intensity and UV-B radiation [67]. The effect of PAR and UV-B on essential oil components is likely due to its effects on modulating the expression of genes involved in phenylpropanoids and terpenoids biosynthetic pathways [60]. A slight change in enzymatic activity may lead to an increase or decrease in certain compounds and make a change in the composition of essential oil [68]. Citronellol and geraniol, as the major constituents of *P. graveolens *essential oil, are alcoholic monoterpenes synthesized by the biosynthetic pathway of methylerythritol-4-phosphate (MEP) [64, 65]. The precursor supplies (IPP and DMAPP) is a limiting factor in the biosynthesis of monoterpenes from the plastidial MEP pathway [69]. Considering the fact that monoterpenes are synthesized in plastids, any chloroplast damage induced by UV-B and/or high PAR may lead to change in monoterpene biosynthesis. It has been shown that UV-B altered expression of genes involved in essential oil biosynthesis in peppermint, subsequently made change in essential oil composition [69, 70]. Many publications report the change in essential oil composition under UV-B exposure or PAR intensity. For example, in lemongrass, UV-B exposure increased the essential oil content. Furthermore, the proportion of *Z*-citral, geraniol formate, linalyl formate was also significantly changed following UV-B treatment [60]. Rai and Agrawal [56] also reported significant increment in some medicinally important constituents of *E. alba* essential oil such as *α*-terpineol, δ-cadinene and methyl linoleate exposed to continuous UV-B irradiation. The findings obtained from the study of Raffo et al., [23] on *R. officinalis* demonstrated that the relative percentages of camphene, *α*-pinene, myrcene, 1,8-cineole, *β*-pinene, *α*-terpinene, *β*-caryophyllene and α-phellandrene increased in plants exposed to 50% sunlight, whereas camphor, 3-carene, terpinen-4-ol, borneol, verbenone, *α*-terpineol, and humulene showed decrease with this treatment.

## Conclusions

In this outdoor study, an attempt was made to elucidate whether high PAR intensity can minimize reduction in growth and yield of *P. graveolens* exposed to UV-B irradiation. High PAR intensity reduced the length and width of leaf, the height of plant, as well as fresh weight of aerial parts, but increased dry weight of aerial parts. On the other hand, increase of UV-B irradiation was associated to reduction of plant height, leaf expansion, and fresh and dry weight of aerial parts. The negative effect of UV-B irradiation on the yield was ameliorated by high PAR intensity. Our hypothesis was confirmed based on the data obtained from yield and yield attributes. Enhanced UV-B-induced changes in plant secondary metabolite biosynthesis could be considered as an adaptive response to UV-B irradiance and should be regarded as a crucial element alongside tolerance mechanisms. Both high PAR and enhanced UV-B prompt the accumulation of phenols, flavonoids and essential oil. Considerable changes were also registered not only for citronellol and geraniol as major components of the essential oil, but also for some minor compounds. Interestingly, the citronellol/geraniol ratio was significantly reduced under UV-B radiation and/or high PAR intensity, giving higher economic value to rose geranium essential oil. A positive relationship between high PAR intensity or UV-B irradiation with total phenol, flavonoids, antioxidant activity as well as properties and production of essential oil was observed, which increased the biological activity and economic value of the plant. Overall, we conclude that PAR and UV-B radiation, as two important environmental factors, have positive effects on the quality and quantity of rose-geranium secondary metabolites.

## Data Availability

All the data generated or analyzed during the current study were included in the manuscript. The raw data is available from the corresponding author on reasonable request.

## References

[1] Cockell CS, Knowland J (1999). Ultraviolet radiation screening compounds. Biol Rev.

[2] Del-Castillo-Alonso MÁ, Diago MP, Monforte L, Tardaguila J, Martínez-Abaigar J, Núñez-Olivera E (2015). Effects of UV exclusion on the physiology and phenolic composition of leaves and berries of Vitis vinifera cv. Graciano. J Sci Food Agric.

[3] Kovács V, Gondor OK, Szalai G, Majláth I, Janda T, Pál M (2014). UV-B radiation modifies the acclimation processes to drought or cadmium in wheat. Environ Exp Bot.

[4] Maharaj R. Effects of abiotic stress (UV-C) induced activation of phytochemicals on the postharvest quality of horticultural crops. In: Venketeshwer, R., Leticia, R. (Eds.), Phytochemicals Isolation, Characterisation and Role in Human Health. InTech. 2015;221–244. 10.5772/60050.

[5] Stapleton AE (1992). Ultraviolet radiation and plants: burning questions. Plant Cell.

[6] Frohnmeyer H, Staiger D (2003). Ultraviolet-B radiation-mediated responses in plants. Balancing damage and protection. J Plant Physiol.

[7] Sharma S, Guruprasad KN (2012). Enhancement of root growth and nitrogen fixation in *Trigonella* by UV-exclusion from solar radiation. Plant Physiol Biochem.

[8] Ballare CL, Caldwell MM, Flint SD, Robinson SA, Bornman JF (2011). Effects of solar ultraviolet radiation on terrestrial ecosystems. Patterns, mechanisms, and interactions with climate change. Photochem Photobiol Sci.

[9] Müller V, Lankes C, Albert A, Winkler JB, Zimmermann BF, Noga G, Hunsche M (2015). Concentration of hinokinin, phenolic acids and flavonols in leaves and stems of *Hydrocotyle leucocephala* is differently influenced by PAR and ecologically relevant UV-B level. J Plant Physiol.

[10] Vidović M, Morina F, Milić S, Albert A, Zechmann B, Tosti T, Jovanović SV (2015). Carbon allocation from source to sink leaf tissue in relation to flavonoid biosynthesis in variegated *Pelargonium zonale* under UV-B radiation and high PAR intensity. Plant Physiol Biochem.

[11] Shayganfar A, Azizi M, Rasouli M (2018). Various strategies elicited and modulated by elevated UV-B radiation and protectant compounds in *Thymus* species: Differences in response over treatments, acclimation and interaction. Ind Crops Prod.

[12] Brune D, Hellborg R, Persson BRR, Pääkkönen R (2003). Radiation: At Home, Outdoors and in the Workplace. Am J Phys.

[13] Verdaguer D, Jansen MAK, Llorens L, Morales LO, Neugart S (2017). UV-A radiation effects on higher plants: Exploring the known unknown. Plant Sci.

[14] Liakoura V, Bornman JF, Karabourniotis G (2003). The ability of abaxial and adaxial epidermis of sun and shade leaves to attenuate UV-A and UV-B radiation in relation to the UV absorbing capacity of the whole leaf methanolic extracts. Physiol Plant.

[15] Kataria S, Dehariya P, Guruprasad KN, Pandey GP (2012). Effect of exclusion of ambient solar UV-A/B components on growth and antioxidant response of cotton (Gossypium hirsutum L.). Acta biologica Cracoviensia. Ser Bot.

[16] Seckmeyer G, Pissulla D, Glandorf M, Henriques D, Johnsen B, Webb A, Siani AM, Bais A, Kjeldstad B, Brogniez C (2008). Variability of UV irradiance in Europe. Photochem Photobiol.

[17] Krizek DT, Chalker-Scott L (2005). Ultraviolet radiation and terrestrial ecosystems. Photochem Photobiol.

[18] Mumivand H, Shayganfar A, Tsaniklidis G, Emami Bistgani Z, Fanourakis D, Nicola S (2021). Pheno-morphological and essential oil composition responses to UVA radiation and protectants: A case study in three Thymus species. Horticulturae.

[19] Flint SD, Caldwell MM (1996). Scaling plant ultraviolet spectral responses from laboratory action spectra to field spectral weighting factors. J Plant Physiol.

[20] Kataria S, Baroniya SS, Kanungo M, Bhaghel L (2014). Effect of Exclusion of Solar UV radiation on Plants. Plant Science Today.

[21] Shiozaki N, Hattori I, Gojo R, Tezuka T (1999). Activation of growth and nodulation in a symbiotic system between pea plants and leguminous bacteria by near-UV radiation. J Photochem Photobiol A Chem B Biol.

[22] Helsper JPFG, Ric de Vos CH, Maas FM, Jonker HH, Van Den Broeck HC, Jordi W, Pot CS, Keizer LCP, Schapendonk AHCM (2003). Response of selected antioxidants and pigments in tissues of *Rosa hybrida* and *Fuchsia hybrida* to supplemental UV-A exposure. Physiologia Plantarum.

[23] Raffo A, Mozzanini E, Ferrari Nicoli S, Lupotto E, Cervelli C (2020). Effect of light intensity and water availability on plant growth, essential oil production and composition in *Rosmarinus officinalis* L. Eur Food Res Technol.

[24] Matsuura HN, Costa FD, Yendo ACA, Fett-Neto AG. Photoelicitation of bioactive secondary metabolites by ultraviolet radiation: mechanisms, strategies, and applications. Biotechnol Med Plants. 2013;171–190. 10.1007/978-3-642-29974-2_7.

[25] Escobar AL, De Oliveira Silva FM, Acevedo P, Nunes-Nesi A, Alberdi M, Reyes-Diaz M (2017). Different levels of UV-B resistance in *Vaccinium corymbosum* cultivars reveal distinct backgrounds of phenylpropanoid metabolites. Plant Physiology Biochem.

[26] Bolink EM, Van Schalkwijk I, Posthumus F, Van Hasselt PR (2001). Growth under UV-B radiation increases tolerance to high-light stress in pea and bean plants. Plant Ecol.

[27] Behn H, Albert A, Marx F, Noga G, Ulbrich A (2010). Ultraviolet-B and photosynthetically active radiation interactively affect yield and pattern of monoterpenes in leaves of peppermint (Mentha piperita L.). J Agric Food Chem.

[28] Majer P, Hideg E (2012). Existing antioxidant levels are more important in acclimation to supplemental UV-B irradiation than inducible ones: studies with high light pretreated tobacco leaves. Emir J Food Agric.

[29] Amiri R, Nikbakht A, Etemadi N (2015). Alleviation of drought stress on rose geranium [Pelargonium graveolens (L.) Herit.] in terms of antioxidant activity and secondary metabolites by mycorrhizal inoculation. Scientia Horticulturae.

[30] Rao BR (2002). Biomass yield, essential oil yield and essential oil composition of rose-scented geranium (Pelargonium species) as influenced by row spacings and intercropping with cornmint (Mentha arvensis L.f. piperascens Malinv. ex Holmes). Industrial Crops and Products..

[31] Rezaei Nejad A, Izadi Z, Sepahvand K, Mumivand H, Mousavifard S (2020). Changes in total phenol and some enzymatic and non-enzymatic antioxidant activities of rose-scented geranium (*Pelargonium graveolens*) in response to exogenous ascorbic acid and iron nutrition. JOP.

[32] Ćavar S, Maksimović M (2012). Antioxidant activity of essential oil and aqueous extract of *Pelargonium graveolens* L. Her Food Controlr.

[33] Patel A, Patra DD (2015). Phytoextraction capacity of Pelargonium graveolens L. Hér. grown on soil amended with tannery sludge-Its effect on the antioxidant activity and oil yield. Ecol Eng.

[34] Boukhatem MN, Kameli A, Saidi F (2013). Essential oil of Algerian rose-scented geranium (*Pelargonium graveolens*): Chemical composition and antimicrobial activity against food spoilage pathogens. Food Control.

[35] Benelli G, Pavela R, Canale A, Cianfaglione K, Ciaschetti G, Conti F, Maggi F (2017). Acute larvicidal toxicity of five essential oils (*Pinus nigra*, *Hyssopus officinalis*, *Satureja montana*, *Aloysia citrodora* and *Pelargonium graveolens*) against the filariasis vector Culex quinquefasciatus: Synergistic and antagonistic effects. Parasitol Int.

[36] Rosato A, Maggi F, Cianfaglione K, Conti F, Ciaschetti G, Rakotosaona R, Corbo F (2018). Chemical composition and antibacterial activity of seven uncommon essential oils. J Essent Oil Res.

[37] Nooshkam A, Mumivand H, Hadian J, Alemardan A, Morshedloo MR (2017). Drug yield and essential oil and carvacrol contents of two species of Satureja (S. khuzistanica Jamzad and S. rechingeri Jamzad) cultivated in two different locations. J Appl Res Med Aromat Plants.

[38] Bian ZH, Yang QC, Liu WK (2015). Effects of light quality on the accumulation of phytochemicals in vegetables produced in controlled environments: a review. J Sci Food Agric.

[39] Mumivand H, Ebrahimi A, Morshedloo MR, Shayganfar A (2021). Water deficit stress changes in drug yield, antioxidant enzymes activity and essential oil quality and quantity of Tarragon (*Artemisia dracunculus* L.). Ind Crops Prod.

[40] Taheri-Garavand A, Mumivand H, Fanourakis D, Fatahi S, Taghipour S (2021). An artificial neural network approach for non-invasive estimation of essential oil content and composition through considering drying processing factors: A case study in *Mentha aquatica*. Ind Crops Prod.

[41] Beiranvandi M, Akbari N, Ahmadi A, Mumivand H, Nazarian F (2022). Biochar and super absorbent polymer improved growth, yield, and phytochemical characteristics of *Satureja rechingeri* Jamzad in water-deficiency conditions. Ind Crops Prod.

[42] Q-lab. q-lab.com. 2018. Available online: https://www.q-lab.com/products/lamps-optical-filters/lamps-and-optical-filters. Accessed on 16 Oct 2018.

[43] McDonald S, Prenzler PD, Antolovich M, Robards K (2001). Phenolic content and antioxidant activity of olive extracts. Food Chem.

[44] Quettier-Deleu C, Gressier B, Vasseur J, Dine T, Brunet C, Luyckx M, Cazin M, Cazin JC, Bailleul F, Trotin F (2000). Phenolic compounds and antioxidant activities of buckwheat (*Fagopyrum esculentum* Moench) hulls and flour. J Ethnopharmacol.

[45] Choi CW, Kim SC, Hwang SS, Choi BK, Ahn HJ, Lee MY, Kim SK (2002). Antioxidant activity and free radical scavenging capacity between Korean medicinal plants and flavonoids by assay-guided comparison. Plant Sci.

[46] Mumivand H, Shayganfar A, Hasanvand F, Maggi F, Alizadeh A, Darvishnia M (2021). Antimicrobial Activity and Chemical Composition of Essential Oil from *Thymus daenensis* and *Thymus fedtschenkoi* During Phenological Stages. J Essent Oil-Bear Plants.

[47] Hlatshwayo MS, Wahome PK (2010). Effects of shading on growth, flowering and cut flower quality in carnation (*Dianthus caryophyllus*). J Agric Forestry Social Sci.

[48] Klem K, Ač A, Holub P, Kováč D, Špunda V, Robson TM, Urban O (2012). Interactive effects of PAR and UV radiation on the physiology, morphology and leaf optical properties of two barley varieties. Environ Exp Bot.

[49] Givnish TJ (1988). Adaptation to sun and shade: a whole-plant perspective. Funct Plant Biol.

[50] Hui R, Li XR, Jia RL, Liu LC, Zhao RM, Zhao X, Wei YP (2014). Photosynthesis of two moss crusts from the Tengger Desert with contrasting sensitivity to supplementary UV-B radiation. Photosynthetica.

[51] Mumivand H, Izadi Z, Amirizadeh F, Maggi F, Morshedloo MR (2023). Biochar amendment improves growth and the essential oil quality and quantity of peppermint (Mentha× piperita L) grown under waste water and reduces environmental contamination from waste water disposal. J Hazard Mater.

[52] Müller V, Albert A, Winkler JB, Lankes C, Noga G, Hunsche M (2013). Ecologically relevant UV-B dose combined with high PAR intensity distinctly affect plant growth and accumulation of secondary metabolites in leaves of Centella asiatica L Urban. J Photochem Photobiol B.

[53] Burchard P, Bilger W, Weissenböck G (2008). Contribution of hydroxycinnamates and flavonoids to epidermal shielding of UV-A and UV-B radiation in developing rye primary leaves as assessed by ultraviolet-induced chlorophyll fluorescence measurements. Plant, Cell Environ.

[54] Jansen MAK (2002). Ultraviolet-B radiation effects on plants: induction of morphogenic responses. Physiol Plant.

[55] Barbosa SM, do Couto Abreu N, de Oliveira MS, Cruz JN, de Aguiar Andrade EH, Neto MAM, Gurgel ESC (2021). Effects of light intensity on the anatomical structure, secretory structures, histochemistry and essential oil composition of *Aeollanthus suaveolens* Mart. ex Spreng. (Lamiaceae). Biochem Syst Ecol.

[56] Rai K, Agrawal SB (2020). Effect on essential oil components and wedelolactone content of a medicinal plant *Eclipta alba* due to modifications in the growth and morphology under different exposures of ultraviolet-B. Physiol Mol Biol Plants.

[57] Degani AV, Dudai N, Bechar A, Vaknin Y (2016). Shade effects on leaf production and essential oil content and composition of the Novel Herb Eucalyptus citriodora Hook. J Essent Oil-Bear Plants.

[58] Mousavinik SM, Asgharipour MR, Sardashti S (2016). Manure and light intensity affect growth characteristics and essential oil of peppermint (Mentha piperita L.). J Essent Oil-Bear Plants.

[59] Mumivand H, Khanizadeh P, Morshedloo MR, Sierka E, Żuk-Gołaszewska K, Horaczek T, Kalaji HM (2021). Improvement of growth, yield, seed production and phytochemical properties of *Satureja khuzistanica* Jamzad by foliar application of boron and zinc. Plants.

[60] Kumari R, Agrawal SB, Singh S, Dubey NK (2009). Supplemental ultraviolet-B induced changes in essential oil composition and total phenolics of Acorus calamus L. (sweet flag). Ecotoxicol Environ Saf.

[61] Kumari R, Agrawal SB (2011). Comparative analysis of essential oil composition and oil containing glands in Ocimum sanctum L. (Holy basil) under ambient and supplemental level of UV-B through gas chromatography–mass spectrometry and scanning electron microscopy. Acta Physiol Plant.

[62] Wang D, Chen L, Ma Y, Zhang M, Zhao Y, Zha X (2019). Effect of UV-C treatment on the quality of fresh-cut lotus (Nelumbo nucifera Gaertn.) root. Food Chem.

[63] Morshedloo MR, Amani Machiani M, Mohammadi A, Maggi F, Aghdam MS, Mumivand H, Javanmard A (2021). Comparison of drying methods for the extraction of essential oil from dragonhead (Dracocephalum moldavica L., Lamiaceae). J Essent Oil Res.

[64] Saxena G, Laiq-ur-Rahman Verma PC, Banerjee S, Kumar S (2008). Field performance of somaclones of rose scented geranium (*Pelargonium graveolens* L’Her Ex Ait) for evaluation of their essential oil yield and composition. Ind Crops Prod.

[65] Verma RK, Rahman LU, Verma RS, Kalra A, Kumar A, Kukreja A, Bisht AS, Chauhan A, Khanuja SPS (2010). Assessing N-us efficiency, planting time and economics of fertilizer N in rose-scented geranium (*Pelargonium graveolens* L’Herit) in Western Himalayan Region of India. Afr J Agric Res.

[66] Peterson A, Machmudah S, Roy BC, Goto M, Sasaki M, Tsutomu H (2006). Extraction of essential oil from geranium (*Pelargonium graveolens*) with supercritical carbon dioxide. J Chem Technol Biotechnol.

[67] Manukyan A (2013). Effects of PAR and UV-B radiation on herbal yield, bioactive compounds and their antioxidant capacity of some medicinal plants under controlled environmental conditions. Photochem Photobiol.

[68] Figueiredo AC, Barroso JG, Pedro LG, Scheffer JJC (2008). Factors affecting secondary metabolite production in plants: volatile components and essential oils. Flavour Fragr J.

[69] Dolzhenko Y, Bertea CM, Occhipinti A, Bossi S, Maffei ME (2010). UV-B modulates the interplay between terpenoids and flavonoids in peppermint (Mentha× piperita L.). J Photochem Photobiol B.

[70] Alagupalamuthirsolai M, Ankegowda SJ, Murugan M, Sivaranjani R, Rajkumar B, Akshitha HJ (2019). Influence of light intensity on photosynthesis, capsule yield, essential oil and insect pest incidence of small cardamom (Elettaria cardamomum (L.) Maton). J Essent Oil-Bear Plant.

